# N^6^-methyladenosine modification of viral RNA and its role during the recognition process of RIG-I-like receptors

**DOI:** 10.3389/fimmu.2022.1031200

**Published:** 2022-12-13

**Authors:** Huanan Li, Yang Guo, Wenbao Qi, Ming Liao

**Affiliations:** ^1^ National Avian Influenza Para-Reference Laboratory (Guangzhou), South China Agricultural University, Guangzhou, China; ^2^ Key Laboratory of Zoonosis, Ministry of Agriculture and Rural Affairs, Guangzhou, China; ^3^ National and Regional Joint Engineering Laboratory for Medicament of Zoonoses Prevention and Control, Guangzhou, China; ^4^ Key Laboratory of Zoonosis Prevention and Control of Guangdong Province, Guangzhou, China; ^5^ Guangdong Laboratory for Lingnan Modern Agriculture, Guangzhou, China; ^6^ Guangdong Academy of Agricultural Sciences, Guangzhou, China

**Keywords:** m^6^A, viruses, replication, RIG-I-like receptors, innate immune escape

## Abstract

N^6^‐methyladenosine (m^6^A) is the most abundant RNA chemical modification in eukaryotes and is also found in the RNAs of many viruses. In recent years, m^6^A RNA modification has been reported to have a role not only in the replication of numerous viruses but also in the innate immune escape process. In this review, we describe the viruses that contain m^6^A in their genomes or messenger RNAs (mRNAs), and summarize the effects of m^6^A on the replication of different viruses. We also discuss how m^6^A modification helps viral RNAs escape recognition by exogenous RNA sensors, such as retinoic acid-inducible gene I (RIG-I)-like receptors (RLRs), during viral invasion. Overall, the goal of our review is to summarize how m^6^A regulates viral replication and facilitates innate immune escape. Furthermore, we elaborate on the potential of m^6^A as a novel antiviral target.

## Introduction

N^6^-methyladenosine (m^6^A) was the first internal RNA modification identified in mRNAs of mammalian cells in 1974 (1). However, our understanding of m^6^A is currently limited. In 1994, 20 years after the discovery of m^6^A, methyltransferase was identified as a protein complex, and methyltransferase-like 3 (METTL3) was identified as an S−adenosyl methionine (SAM)-binding protein with methyltransferase capacity (2, 3). M^6^A is the most abundant and well-characterized RNA modification (4, 5). Additionally, m^6^A is a reversible chemical modification that affects nearly all aspects of RNA biology, including RNA folding structure, mRNA maturation, nuclear export, translation, and mRNA decay (6–20).

M^6^A was once termed as the fifth base in mRNA. It was first identified by chromatography (1). Methylated RNA immunoprecipitation sequencing (MeRIP-seq), also named m^6^A-seq, is the most widely used sequencing method for RNA m^6^A profiling, but it cannot precisely identify which adenosines are modified (21). M^6^A individual-nucleotide resolution crosslinking and immunoprecipitation sequencing (miCLIP-m6A-seq) and photo-crosslinking-assisted m^6^A sequencing (PA-m6A-seq) can detect and characterize m^6^A in RNA with pinpoint accuracy (22). In addition to mRNA of mammalian cells, m^6^A has been identified in a wide range of viral RNAs, including DNA and RNA viruses (as shown in **Table 1**). The replication of many viruses can be modulated by m^6^A, and in-depth studies have revealed that m^6^A exhibits contrary functions in the replication process of different viruses. Additionally, m^6^A affects the recognition of viral RNAs by RLRs (38–40).

**Table 1:** Effect of m^6^A on DNA virus.

Virus	Genome	Effect of m^6^A on virus replication	Reference	Effect of m^6^A on RLR sensing	Reference
Kaposi's sarcoma‐associated	double-stranded DNA	promote virus reproduction in iSLK.219 cells, iSLK.BAC16 cells and B cells	(23, 24)	no applicable data found	no applicable data found
herpesvirus (KSHV)		suppress virus reproduction in KiSLK cells	(25)		
		suppress virus reproduction in TREx BCBL1-Rta cells	(26)		
Epstein–Barr virus (EBV)	double-stranded DNA	promote virus reproduction	(27, 28)	no applicable data found	no applicable data found
		suppress virus reproduction	(29)		
Herpes simplex virus 1 (HSV‐1)	double-stranded DNA	promote virus reproduction	(30)	no applicable data found	no applicable data found
Simian vacuolating virus 40 (SV40)	double-stranded DNA	promote virus reproduction	(31)	no applicable data found	no applicable data found
Adenovirus (AdV)	double-stranded DNA	promote virus reproduction	(32)	no applicable data found	no applicable data found
*Bombyx mori* nucleopolyhedrovirus (BmNPV)	circular double-stranded DNA	suppress virus reproduction	(33)	no applicable data found	no applicable data found
Human Papillomaviruse 16 (HPV-16)	circular double-stranded DNA	promote virus reproduction	(34)	no applicable data found	no applicable data found
Hepatitis B virus (HBV)	partially double-stranded DNA	promote virus reproduction	(35, 36)	attenuate RIG-I sensing activity	(38)
		suppress virus reproduction	(35, 37)		

Innate immune responses function as the primary antiviral strategy when host cells are invaded by viruses. RLRs are key sensors among pattern recognition receptors (PRRs). RLRs can recognize exogenous viral RNAs and stimulate the production of type I interferons (IFNs), which can result in the upregulation of antiviral proteins, such as RNA-dependent protein kinase (PKR), 2′,5′-oligoadenylate synthetase (OAS), 2’,5’-oligoadenylate-dependent ribonuclease L (RNase L), and Mx proteins (41, 42). RLRs include three components: RIG-I, melanoma differentiation-associated protein 5 (MDA5), and laboratory of genetics and physiology 2 (LGP2) (43, 44). RIG-I recognizes double-stranded RNAs (dsRNAs) (<300 bp) containing either a 5’-triphosphate or 5’-diphosphate (45–47); MDA5, which shares a similar structure with RIG-I, senses long dsRNAs (>1,000 bp) (48, 49); and LGP2, which lacks the caspase recruitment domain (CARD), is a regulator of RIG-I and MDA5, and exhibits different regulatory functions (50–52). RIG-I and MDA5, which are sensors of exogenous viral RNAs, can sense RNAs generated by both DNA and RNA viruses.

Interestingly, m^6^A modifications in the RNAs of different viruses exhibit many differences during the replication process. Moreover, m^6^A modifications in viral RNAs play a significant role in RLR recognition after viral infection. Here, we review the function of m^6^A in viral replication and the innate immune sensing of RLRs.

## M^6^A RNA methylation

**Figure 1:**
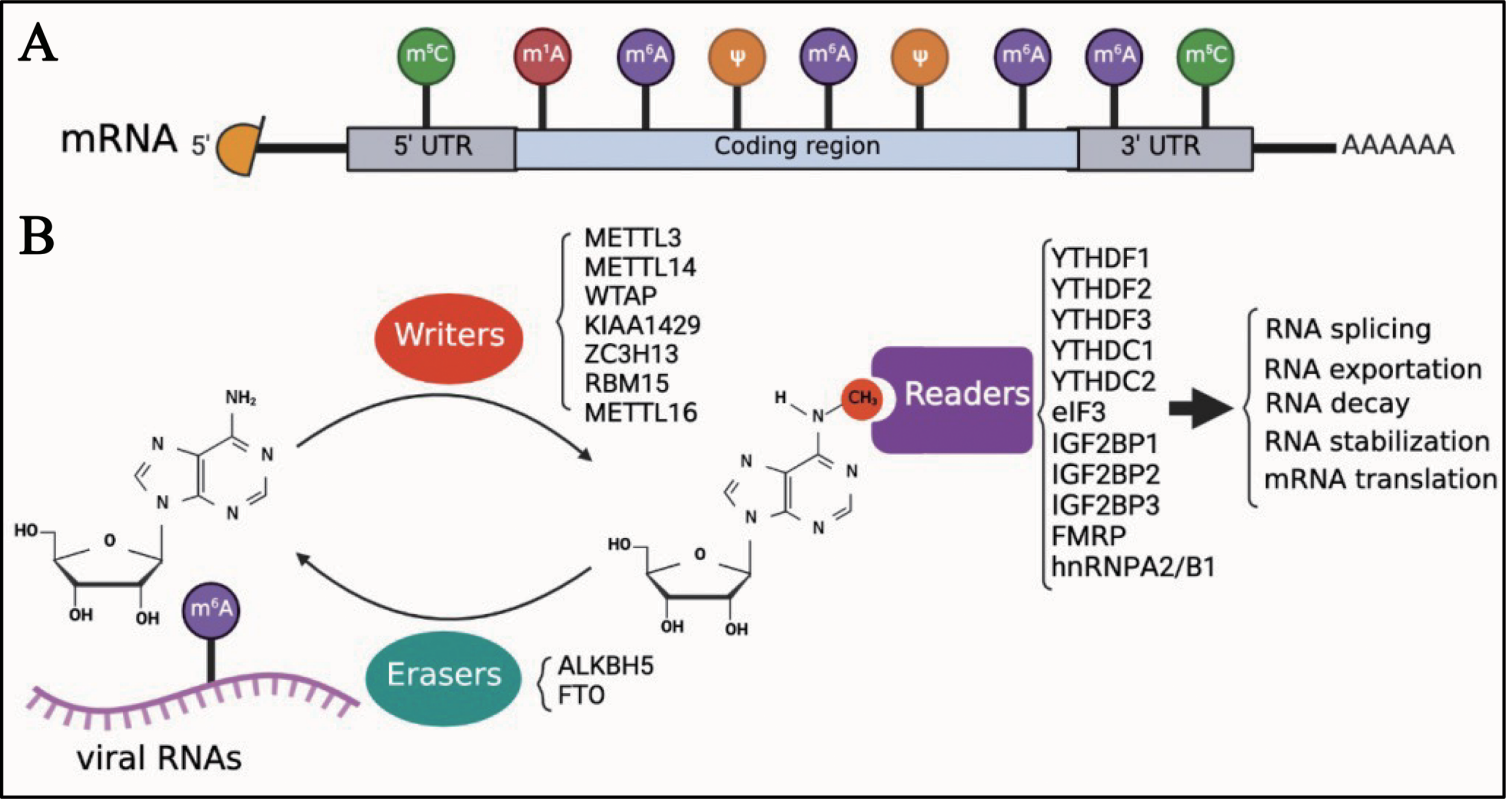
Introduction of m^6^A modifications. **(A)** M^6^A is one of the first identified and most abundant internal modifications in the mRNA of eukaryotic cells. **(B)** M^6^A is a reversible chemical modification in RNAs; ‘writers’ add a methyl to the N6 position of adenosine; ‘erasers’ remove the methyl of m^6^A; after adding m^6^A in RNAs, ‘readers’ recognize the modified RNAs and regulate the biological process of RNAs, including translation, decay, and translocation.

Eukaryotic cell mRNA has many internal chemical modifications, including m^6^A, 5-methylcytosine (m^5^C), N1-methyladenosine (m^1^A), and pseudouridine (Ψ) (1, 53–56); among these, m^6^A is the most abundant modification (**Figure 1A**). In addition, m^6^A is a reversible chemical modification (**Figure 1B**). The RNA transferases, including METTL3, methyltransferase-like 14 (METTL14), WT1-associated protein (WTAP), KIAA1429 (also known as vir-like m^6^A methyltransferase-associated protein [VIRMA]), zinc finger CCCH domain-containing protein 1 (ZC3H13), RNA-binding motif protein 15 (RBM15), and methyltransferase-like 16 (METTL16) are termed as ‘writers’. METTL3, METTL14, and WTAP, which are the most well-known ‘writers’, can form a protein complex. This protein complex can recognize the consensus DRA*CH ([A/G/U], [A/G], A*, C, [A/C/U]) motifs and add a methyl to the specific N6 position of adenosine (22, 57). As previously mentioned, AlkB homolog 5 (ALKBH5) and fat mass and obesity-associated protein (FTO), which function as demethylases, are known as ‘erasers’, and can remove the methyl of m^6^A. ‘Readers’, including YTH N^6^-methyladenosine RNA-binding protein 1, 2, and 3 (YTHDF1, YTHDF2, and YTHDF3), YTH Domain Containing 1 and 2 (YTHDC1 and YTHDC2), eukaryotic initiation factor 3 (eIF3), insulin-like growth factor 2 mRNA-binding protein 1, 2, and 3 (IGF2BP1, IGF2BP2, and IGF2BP3), fragile X mental retardation protein (FMRP), and heterogeneous nuclear ribonucleoproteins A2/B1 (hnRNPA2/B1), recognize the m^6^A modifications in RNAs and regulate several biological processes of RNAs, such as translation, decay, and translocation. Owing to the lack of research techniques, there was limited knowledge about the function of m^6^A until the Chinese-American scientist Chuan He proposed the concept of RNA epigenetics in the early 2010s (58). Since then, significant progress has been made in the study of m^6^A modification. In addition to eukaryotic mRNAs, m^6^A is also found in many viral mRNAs, viral genomes, and intermediate RNAs produced during the viral replication process. In 1976, only 2 years after m6A was identified in eukaryotic mRNAs, influenza virus mRNA was found to contain internal m^6^A modifications (59). Although a few articles on m^6^A modifications of viral RNAs have been published to date, it seems that the study of viral RNA epigenetics is poised for a major expansion and has the potential to change our understanding of how viruses regulate their life cycle.

## Function of m^6^A RNA methylation in the life cycle of DNA viruses

**Figure 2:**
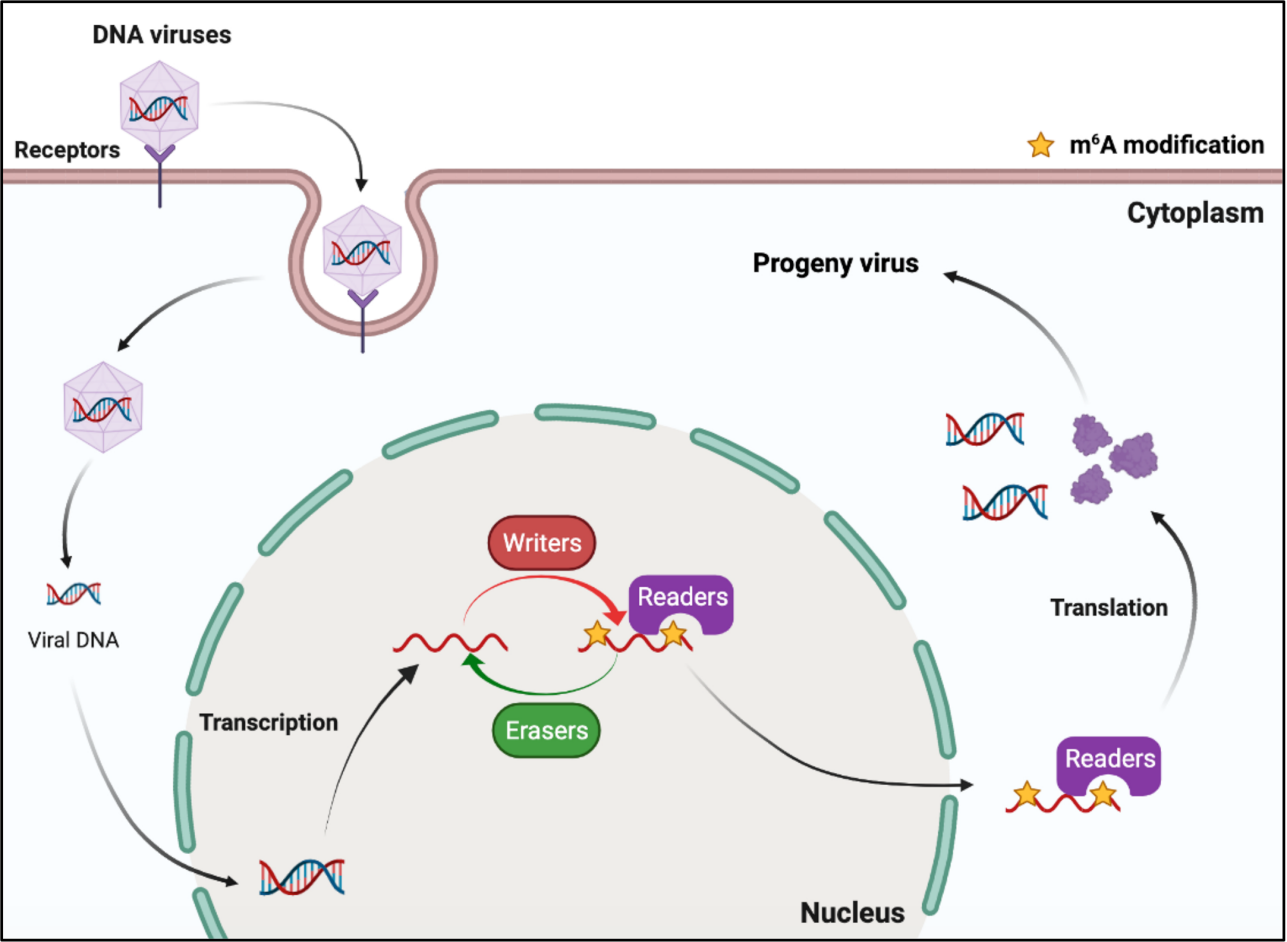
M^6^A modifications of viral RNAs during the replication process of DNA viruses. DNA viruses produce mRNAs during replication. The viral mRNAs can be m^6^A modified, and these modifications can modulate the replication of DNA viruses.

DNA viruses (with DNA genomes) can also produce RNAs (which contain m^6^A) during replication (**Figure 2**). Further studies have shown that m^6^A modifications have different regulatory functions in the life cycle of different viruses (**Table 1**). M^6^A in viral RNAs promotes the replication of herpes simplex virus 1 (HSV‐1), simian vacuolating virus 40 (SV40), adenovirus (AdV), and human papillomavirus 16 (HPV-16) (30–32, 34). However, m^6^A functions as a suppressive regulator of *Bombyx mori* nucleopolyhedrovirus (BmNPV) replication (33). Furthermore, m^6^A in the RNA of Kaposi’s sarcoma‐associated herpesvirus (KSHV) adversely affects replication in different cells. Interestingly, m^6^A functions as a positive regulator of KSHV in iSLK.219, iSLK.BAC16, and B cells, but as a negative regulator in KiSLK and TREx BCBL1-Rta cells (23–26). Different researchers have different opinions regarding the function of m^6^A. M^6^A functions adversely during the replication process of Epstein–Barr virus (EBV) and hepatitis B virus (HBV) (27, 28, 35–37). Although there is no direct sequencing evidence to prove the presence of m^6^A in the RNA of human cytomegalovirus (HCMV), METTL3 and METTL14 small interfering RNAs (siRNAs) inhibit HCMV reproduction, indicating that m^6^A may function as a positive regulator of the life cycle of HCMV (60, 61).

## Function of m^6^A RNA methylation in the life cycle of cytoplasmic RNA viruses

**Figure 3:**
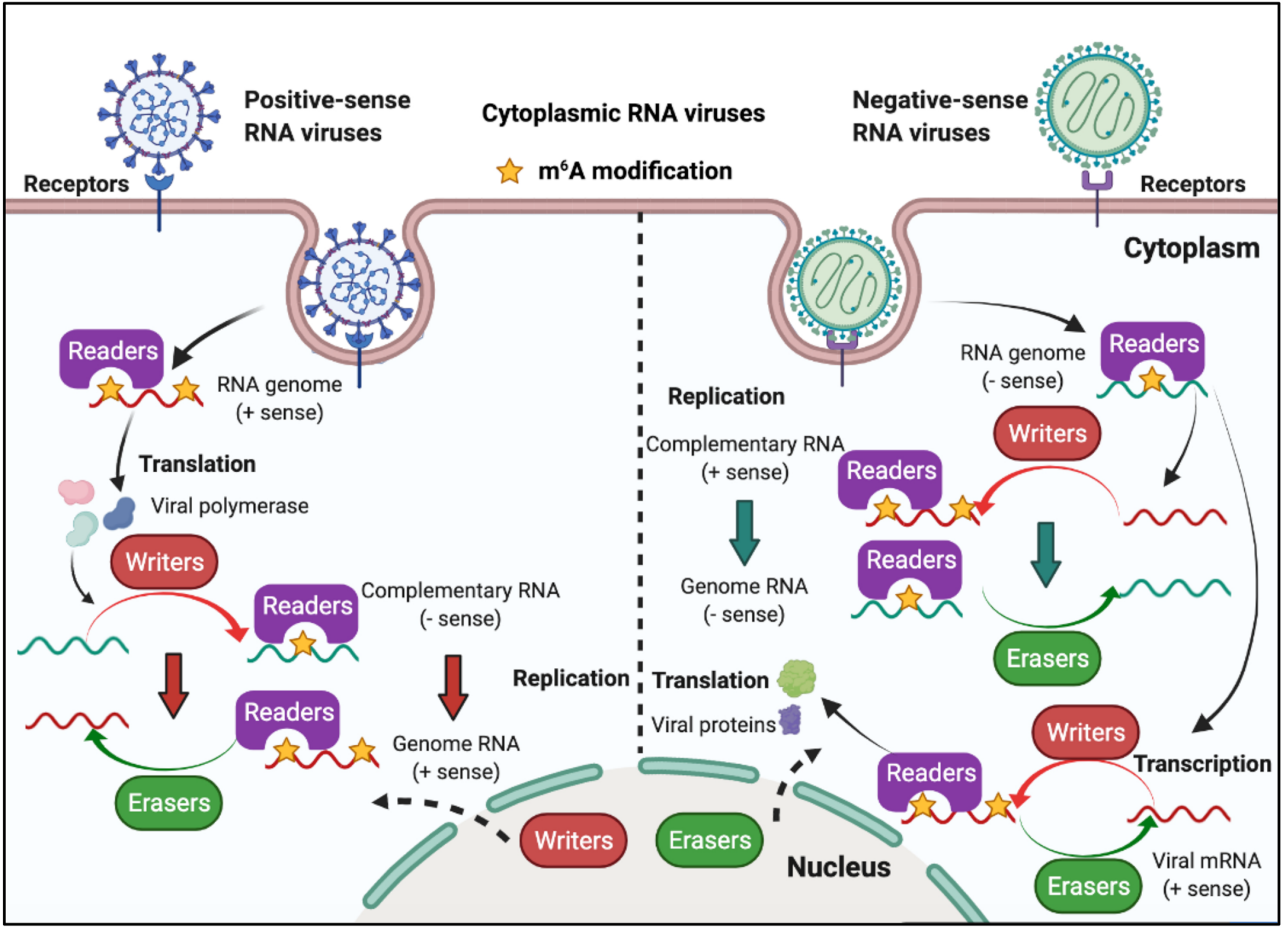
M^6^A modifications of viral RNAs during the replication process of cytoplasmic RNA viruses. Some of the cytoplasmic RNA viruses possess a positive RNA genome, whereas some possess a negative RNA genome. Cytoplasmic RNA viruses can produce mRNA (the positive-sense RNA genome of cytoplasmic RNA viruses functions as mRNA) and cRNA. M^6^A is present in the viral genome RNA, cRNA, and mRNA, and can modulate viral protein expression and viral replication. ‘Writers’ and ‘erasers’ can translocate from the nucleus to the cytoplasm after viral infection.

RNA viruses have RNA genomes, most of which replicate in the cytoplasm. As shown in **Figure 3**, ‘writers’ and ‘erasers’ are located in the nucleus under a steady state; however, they may also be detected in the cytoplasm after infection, suggesting that these proteins can shuttle between the nucleus and the cytoplasm (62–66). The positive-sense RNA genome of cytoplasmic RNA viruses, such as Flaviviridae, Coronavirus, and Picornaviridae, functions as mRNA, and can produce negative-sense complementary RNA (cRNA) by serving as a replication template during the replication process. The genomes of the Pneumoviridate and Rhabdoviridae families consist of negative-sense RNA, and both cRNA and mRNA are produced during transcription. Positive-sense cRNA functions as a template for viral genome replication. M^6^A modifications exist in the viral genome RNA, cRNA, and mRNA of many RNA viruses, and m^6^A plays different roles in different viruses (**Table 2**). Replication of Flaviviridae, including Zika virus (ZIKV), dengue virus (DENV), and hepatitis C virus (HCV), is deeply modulated by m^6^A, and m^6^A in the RNA of these viruses is a suppressive regulator of viral replication (62, 63, 67). With the spread of the SARS-CoV-2 infection since 2019, scientists have been paying great attention to the study of coronaviruses. Liu’s work indicated that both positive and negative RNAs of SARS-CoV-2 contain m^6^A modifications, and m^6^A negatively regulates SARS-CoV-2 infection, as overexpression of METTL3 can inhibit its replication (66, 68–70). Porcine epidemic diarrhea virus (PEDV), a member of the Coronaviridate, also contains m^6^A in its genomic RNA, and m^6^A suppresses its replication (71). Enterovirus 71 (EV71) also possesses a positive-sense RNA genome; however, m^6^A promotes the replication of EV71, which contrasts with its function in the replication of coronavirus. Human respiratory syncytial virus (HRSV, a member of the Pneumoviridae family), human metapneumovirus (HMPV, a member of the Paramyxoviridae family), and vesicular stomatitis virus (VSV, a member of the Rhabdoviridae family) all have negative-sense RNA genomes and share a similar life cycle in the cytoplasm. M^6^A is also found in the genomes of these viruses and plays a positive role in the replication process, as it can promote viral protein expression and help viral RNAs escape RIG-I recognition (72–74).

**Table 2:** Effect of m^6^A on RNA virus.

Virus	Genome	Effect of m^6^A on virus replication	Reference	Effect of m^6^A on RLR sensing	Reference
Zika virus (ZIKV)	positive-sense, single-stranded RNA	suppress virus reproduction	(63)	no applicable data found	no applicable data found
Dengue virus (DENV)	positive-sense, single-stranded RNA	suppress virus reproduction	(67)	no applicable data found	no applicable data found
Hepatitis C virus (HCV)	positive-sense, single-stranded RNA	suppress virus reproduction	(62)	attenuate RIG-I sensing activity	(38)
Severe acute respiratory syndrome coronavirus 2 (SARS-CoV-2)	positive-sense, single-stranded RNA	suppress virus reproduction	(66, 68)	decrease RIG-I binding activity	(69)
Porcine epidemic diarrhea virus (PEDV)	positive-sense, single-stranded RNA	suppress virus reproduction	(71)	no applicable data found	no applicable data found
Enterovirus 71 (EV71)	positive-sense, single-stranded RNA	promote virus reproduction	(64, 65)	no applicable data found	no applicable data found
Human respiratory syncytial virus (HRSV)	negative-sense, single- stranded RNA	promote virus reproduction	(72)	no applicable data found	no applicable data found
Human metapneumovirus (HMPV)	negative-sense, single-stranded RNA	promote virus reproduction	(73)	m^6^A enables viral RNA to escape from RIG-I sensing	(73)
Vesicular stomatitis virus (VSV)	negative-sense, single- stranded RNA	promote virus reproduction	(74)	m^6^A suppress RIG-I sensing *via* reshaping double-stranded RNA	(40, 74)
Influenza A virus (IAV)	segmented, negative-sense, single-stranded RNA	promote virus reproduction	(75)	no applicable data found	no applicable data found
Human immunodeficiency virus type 1 (HIV-1)	two positive-sense, single single-stranded RNA	promote virus reproduction	(76–79)	m^6^A enables viral RNA to escape from RIG-I sensing	(39)
		suppress virus reproduction	(80)		
Endogenous retroviruses (ERVs)	positive-sense, single-stranded RNA	suppress virus reproduction	(81)	no applicable data found	no applicable data found

## Function of m^6^A RNA methylation in the life cycle of intranuclear RNA viruses

**Figure 4:**
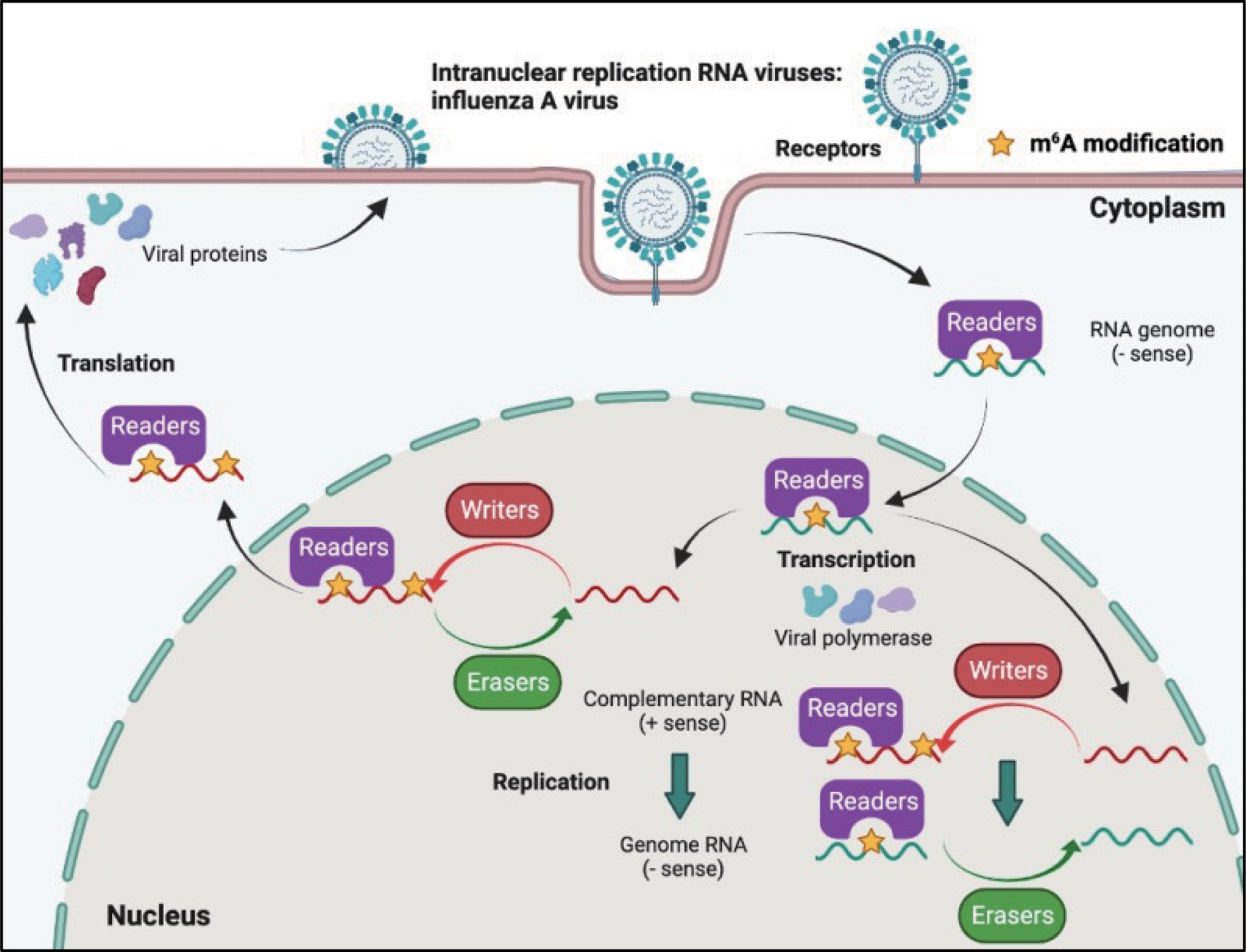
M^6^A modifications of viral RNAs during the replication process of intranuclear RNA viruses. After binding to the receptors, the genome of intranuclear virus enters the nucleus, where replication and transcription occur. M^6^A is present in the viral genome RNA, cRNA, and mRNA. Both RNA translocation and mRNA translation are modulated by m^6^A modification.

Although most RNA viruses replicate in the cytoplasm, for some of them, replication occurs in the nucleus. Influenza virus (belonging to the Orthomyxoviridae family) possesses a segmented negative-sense single-stranded RNA genome, and is a strict intranuclear replication RNA virus. When influenza virus encounters host cells, hemagglutinin (HA) proteins bind to α-(2, 3)-linked or α-(2, 6)-linked sialic acid on the cell surface membrane, and endocytosis of viral particles is triggered. Once influenza virus penetrates the cells, the viral genome can be released from uncoated virion particles and translocated to the nucleus for genome replication; cRNA and mRNA are synthesized in the nucleus during transcription; cRNA acts as a template for the virion RNA (vRNA) replication process, which also occurs in the nucleus; mRNA of the influenza virus is exported to the cytoplasm and serves as a template for the synthesis of viral proteins. Finally, progeny virus assembly and budding is completed in the plasma membrane (**Figure 4**).

The influenza virus was the first confirmed to contain m^6^A, and the replication of influenza virus is modulated by m^6^A (**Table 2**). At first, Krug discovered that m^6^A was present in the mRNA of influenza virus (59); more than 40 years later, Courtney revealed that vRNA and cRNA also contained m^6^A modifications (75). Further studies have indicated that m^6^A promotes influenza replication, and that METTL3 and YTHDF2 play an important role in the replication process (75).

## Function of m^6^A RNA methylation in the life cycle of retroviruses

**Figure 5:**
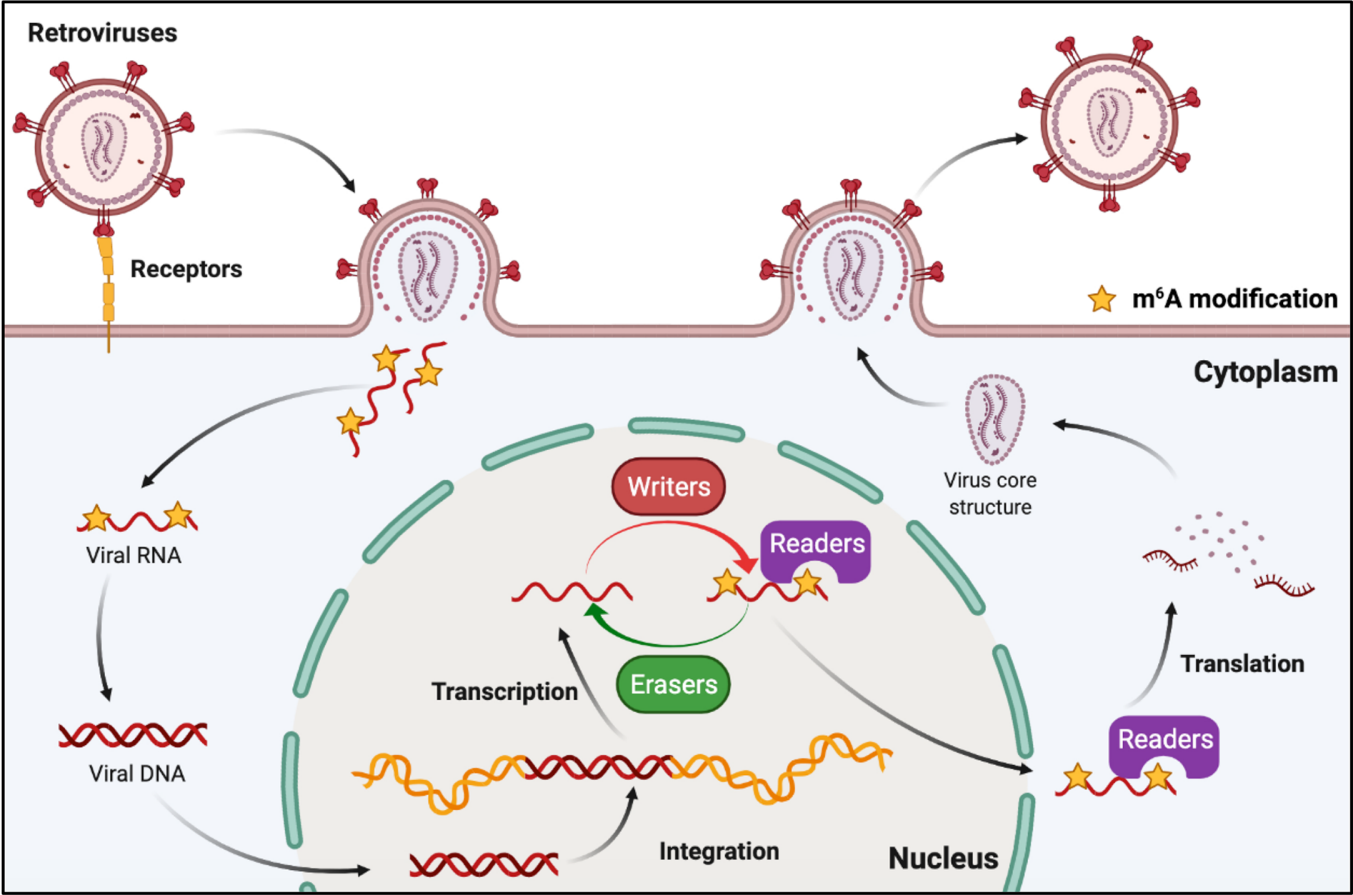
M^6^A modifications of viral RNAs during the replication process of retroviruses. Retroviruses possess an RNA genome and have reverse transcription activity. M^6^A is present in both genomic RNA and mRNA of retroviruses, and plays roles in reverse transcription, RNA transcription, and mRNA translation.

Retroviruses are a family of RNA viruses that have a reverse transcriptase capable of making a complementary DNA copy of the viral genomic RNA, which is then integrated into the host cell’s DNA. M^6^A can also be found in the viral genomic RNA and mRNA of retroviruses (**Figure 5**). Human immunodeficiency virus type 1 (HIV-1), Rous sarcoma virus (RSV), and feline leukemia virus (FeLV) all belong to groups of retroviruses that contain m^6^A modifications in their RNAs (76, 77, 82–84). It was discovered that m^6^A plays different roles in the replication of retroviruses (**Table 2**). Many research groups have revealed that post-transcriptional m^6^A modification of HIV-1 mRNAs enhances viral gene expression, whereas Lu et al. discovered that YTHD proteins could bind to the genomic RNA of HIV-1 and inhibit viral reverse transcription after viral entry (76–80). A recent study has revealed that m^6^A functions as a suppressive regulator of the life cycle of endogenous retroviruses (ERVs). Host cells can recognize m^6^A modifications in the mRNAs of intracisternal A-particles (IAPs) and related ERVK elements, and the m^6^A-modified RNAs can restrain their ability to trigger inflammatory responses, such as those observed in human neurodegenerative diseases (81, 85).

## Function of m^6^A RNA methylation in RLR sensing

**Figure 6:**
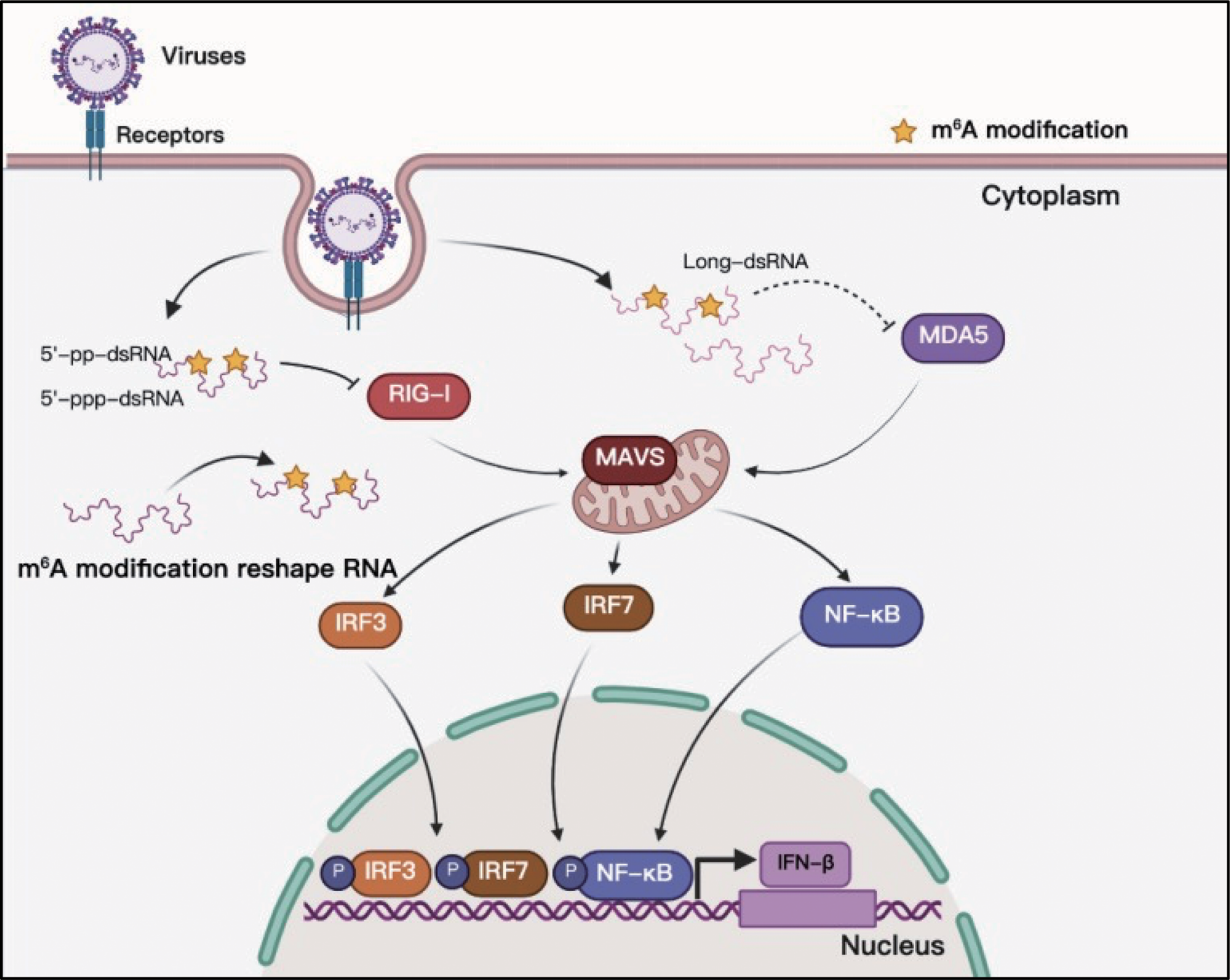
M^6^A modifications of viral RNAs and their function in RLR sensing. Viral RNAs can form complicated secondary structures, and RLRs can recognize the double-stranded component. Adding m^6^A to viral RNAs can reshape RNA structure and enable viral RNAs to escape RLR recognition.

Toll-like receptors (TLRs) and RLRs are the main receptors of PRRs that can sense viral RNAs. Studies of PRR sensing of m^6^A-modified viral RNA have been mainly focused on RLRs. RIG-I and MDA5 are the main sensors of RLRs, and their primary function is to recognize exogenous RNA and stimulate the expression of type I IFNs when host cells are invaded by viruses (86). The RNAs produced in the replication process of both DNA and RNA viruses can be recognized by RLRs, and some studies have indicated that m^6^A modification helps exogenous viral RNA escape recognition by RLRs (**Figure 6**). Although HCMV is a DNA virus, it can trigger RLR sensing activity, and m^6^A might play a key role in this process (60, 87). However, the interaction between m^6^A and RLR stimulation has not yet been thoroughly clarified, and there is no direct evidence to indicate that the mRNA of HCMV contains m^6^A (60). This study suggests that m^6^A might play a role in the recognition of HCMV viral mRNA. HBV is another well-known DNA virus; but its life cycle produces an RNA intermediate termed ‘pregenomic’ RNA (pgRNA). Furthermore, pgRNA is modified by m^6^A, which reduces the sensing activity of RIG-I (38, 88). RLRs mainly recognize viral RNA from RNA viruses during viral infection, and m^6^A in the RNA genomes of HIV-1, HCV, SARS-CoV-2, HMPV, and VSV can help viral RNA escape RIG-I recognition and inhibit the expression of type I IFNs (38, 39, 69, 73, 74). RNAs containing chemically modified nucleotides fail to trigger RLRs, and m^6^A is a functional modification (40, 89, 90). However, the mechanism of how m^6^A-modified RNA escapes RLR sensing remains unclear. Qiu suggested that m^6^A modification impairs the conformation of duplex structures in viral RNAs and interferes with sensing by intracellular receptor RLRs; finally, m^6^A attenuates innate immune response and facilitates immune invasion (74).

## Conclusion and expansion

We have concluded from previous studies that the addition of m^6^A to viral RNAs has both promotive and suppressive functions in the viral life cycle and plays an important role in immune escape from RLRs. M^6^A promotes the replication of DNA viruses, including HSV-1, SV40, AdV, and HPV-16 (30–32, 34), and it has also been shown to positively regulate infection by many RNA viruses, such as EV71, HRSV, HMPV, VSV, and IAV (64, 65, 72–75). By contrast, m^6^A suppresses the replication of DNA viruses, such as BmNPV, and RNA viruses, including ERVs, Flaviviridae, and Coronaviridae (62, 63, 66, 67, 71, 81). Importantly, m^6^A can function as both a proviral and antiviral regulator in the life cycle of some viruses, such as KSHV, EBV, HBV, and HIV-1 (23, 25–28, 35–37, 76–80). The reason why the effect of m^6^A varies between different viruses is uncertain. We think that this is because RNAs of different viruses interact with different ‘readers’.

To date, studies have indicated that m^6^A in viral RNAs could reduce the sensing activity of RLRs and help viruses escape innate immune recognition during viral invasion (38–40, 69, 73, 74). However, other studies have revealed that viral invasion can cause changes in the expression of ‘writers’, ‘readers’, or ‘erasers’, resulting in expression changes in immunoregulatory proteins and eventually influencing IFN production. M^6^A targeting of IFN-β can enhance the destabilization of IFN-β mRNA and restrict the duration of the antiviral response (61). Degradation of WTAP induced by viral infection reduces the m^6^A levels of interferon-regulatory factor 3 (IRF3) and interferon α/β receptor subunit 1 (IFNAR1) mRNAs, resulting in the suppression of IRF3 translation and destabilization of IFNAR1 mRNA (91). Kastan’s work revealed that the RNA-binding protein YTHDF3 promotes the production of interferon-stimulated genes (ISGs); however, Zhang’s work indicated that YTHDF3 functions as a negative regulator of antiviral immunity by promoting the translation of FOXO3 mRNA (92, 93). METTL3, METTL14, and YTHDF1 promote the expression of interferon-induced transmembrane 1 (IFITM1), a well-known ISG (94). A recent study by You’s group reported that m^6^A can stabilize IRF3 mRNA, and Zhu’s group demonstrated that m^6^A can increase the stability of interferon-regulatory factor 7 (IRF7) mRNA (95, 96). As a result, the expression of type I IFNs is enhanced. Therefore, m^6^A has multiple functions in the viral replication process and modulates the antiviral response of type I IFNs.

## Future perspective: m^6^A as a target for antiviral therapy

As m^6^A is present in the life cycle of many viruses, drugs targeting this pathway may have the potential to act as antiviral drugs. For example, 3-deazaadenosine (DAA), an m^6^A modification inhibitor, inhibits the replication of various viruses *in vitro* or *in vivo*, including HRSV, parainfluenza virus type 3 (PIV3), Ebola virus, HIV, and IAV (75, 76, 97, 98). The SARS-CoV-2 pandemic is still ongoing, and studies have provided a proof of concept suggesting that targeting of the cellular components of the m^6^A RNA modification pathway could lead to novel therapeutic opportunities to control this viral pathogen.

In general, the study of viral m6A epitranscriptomics, which started in the early 1970s, has rapidly evolved in the past 5 years, and indicates that m^6^A modification is an important component in viral infections and innate immunity recognition. Importantly, there is a need for a clear mechanistic understanding of m^6^A modifications in viral RNAs to determine their function in viral replication, and to explore their potential as antiviral targets.

## Author contributions

HL, ML, and WQ conceptualized the review. HL and YG wrote the manuscript. HL, WQ, and ML revised the manuscript. All authors contributed to the article and approved the submitted version.
